# A New Nasal Douche

**Published:** 1890-07-05

**Authors:** 


### THE PRACTITIONER'S MIRROR AND RETROSPECT.

[*The Editor will be glad to receive offers of co-operation and contributions from members of the profession. There is no doubt that much valuable material full of interest and instruction is at present lost to science. This is largely so in the case of* (1) *the younger and less-known members of the hospital staff* (2) *those connected with cottage hospitals, and* (3) *the great body of medical practitioners not attached to any institution. The co-operation of all is cordially invited to enable the Editor to make* THE HOSPITAL *of the utmost possible use and value to the profession. Specialists willing to review books on their special subjects are invited to communicate this fact at once. All letters should be addressed to* THE EDITOR, THE LODGE, PORCHESTER SQUARE, LONDON, W.]

### MEDICINE.

### A NEW NASAL DOUCHE.

Apart from the cumbersome size and inconveniences attending the use of most apparatus hitherto employed for washing out or irrigating the nasal passages, there is the possible danger, if too much force be exerted, of driving the fluid into the frontal sinuses or eustachian tubes, to say nothing of the frequent choking or coughing caused by the passage of the water downwards to the glottis, the contraction of the soft palate and fauces excited by the contact of the fluid with their upper and posterior surfaces, being often insufficient to withstand the pressure exerted by it.

Dr. E. Pins, of Vienna, says that he has completely overcome these objections by a simple and handy little contrivance. It consists of a bottle, with an indiarubber cork perforated for two tubes, the larger of which, reaching to the bottom, terminates above in an olive-shaped nozzle to fit the nostril, while the other, which is smaller and too short to reach the surface of the fluid, is connected above to a bent or flexible tube ending in a mouthpiece. Having adapted the nozzle to one nostril and taken the mouthpiece firmly, the patient, with cheeks distended, blows forcibly into the bottle, compressing the contained air, and driving the fluid up the nose-tube. The pressure exerted cannot exceed that of a forced expiration, but the special advantage presented by this method is, that the soft palate, instead of being simply stimulated to contraction by the contact of the fluid above, is forced upwards, and supported by the resistance met with by the expired air in the bottle, thus most effectually closing the nasopharynx. With intervals for deep inspiration, one or two litres may thus be passed through the nose in a few minutes, and children willingly perform the operation themselves. It might, however, be inexpedient in the presence of pulmonary or cardiac disease. In injecting cocaine or other solutions for nasal catarrh, when the mere local application of the drug, and not the flushing out of the passages is aimed at, we have for some years used a small syringe contrived by ourselves, which we find more convenient and less wasteful than the miniature spray. It is a glass U tube of about ⅛-inch inside diameter, the legs a little more than an inch in length and somewhat less than an inch apart; to one is attached a "blind" or imperforate indiarubber teat, and the other is drawn to a fine but blunt point with a capillary aperture. Any desired quantity of the fluid up to 20 minims can be injected with sufficient force to reach every part of the nares, though not more than five to ten drops should be used, since that is the most that can be retained.

